# The International Mouse Phenotyping Consortium Web Portal, a unified point of access for knockout mice and related phenotyping data

**DOI:** 10.1093/nar/gkt977

**Published:** 2013-11-03

**Authors:** Gautier Koscielny, Gagarine Yaikhom, Vivek Iyer, Terrence F. Meehan, Hugh Morgan, Julian Atienza-Herrero, Andrew Blake, Chao-Kung Chen, Richard Easty, Armida Di Fenza, Tanja Fiegel, Mark Grifiths, Alan Horne, Natasha A. Karp, Natalja Kurbatova, Jeremy C. Mason, Peter Matthews, Darren J. Oakley, Asfand Qazi, Jack Regnart, Ahmad Retha, Luis A. Santos, Duncan J. Sneddon, Jonathan Warren, Henrik Westerberg, Robert J. Wilson, David G. Melvin, Damian Smedley, Steve D. M. Brown, Paul Flicek, William C. Skarnes, Ann-Marie Mallon, Helen Parkinson

**Affiliations:** ^1^European Molecular Biology Laboratory, European Bioinformatics Institute (EMBL-EBI), Wellcome Trust Genome Campus, Hinxton, Cambridge CB10 1SD, UK, ^2^Medical Research Council Harwell (Mammalian Genetics Unit and Mary Lyon Centre), Harwell, Oxfordshire OX11 0RD, UK and ^3^Mouse Informatics Group, Wellcome Trust Sanger Institute, Wellcome Trust Genome Campus, Hinxton, Cambridge CB10 1SA, UK

## Abstract

The International Mouse Phenotyping Consortium (IMPC) web portal (http://www.mousephenotype.org) provides the biomedical community with a unified point of access to mutant mice and rich collection of related emerging and existing mouse phenotype data. IMPC mouse clinics worldwide follow rigorous highly structured and standardized protocols for the experimentation, collection and dissemination of data. Dedicated ‘data wranglers’ work with each phenotyping center to collate data and perform quality control of data. An automated statistical analysis pipeline has been developed to identify knockout strains with a significant change in the phenotype parameters. Annotation with biomedical ontologies allows biologists and clinicians to easily find mouse strains with phenotypic traits relevant to their research. Data integration with other resources will provide insights into mammalian gene function and human disease. As phenotype data become available for every gene in the mouse, the IMPC web portal will become an invaluable tool for researchers studying the genetic contributions of genes to human diseases.

## INTRODUCTION

The goal of the International Mouse Phenotyping Consortium (IMPC) is to generate and phenotypically characterize knockout mutant strains for every protein-coding gene in the mouse (1,2). The IMPC was established as a large-scale coordinated effort of mouse clinics worldwide to undertake broad-based primary phenotyping of mutant mouse strains that carry a null mutation in a protein-coding gene (3,4). This program builds on the collection of mutant embryonic stem (ES) cells available from the International Knockout Mouse Consortium (IKMC) (5) and pilot programs that have established a set of robust high-throughput phenotyping assays (6,7). In this article, we describe the functionality and data available from the IMPC web portal delivered by the Mouse Phenotyping Informatics Infrastructure (MPI2) consortium comprising EMBL-EBI, MRC Harwell and the Wellcome Trust Sanger Institute (8).

The IMPC portal is the central point of access to high-throughput phenotype data, IKMC ES cell resources and mutant mouse strains. The progress of mouse production and phenotyping is presented for each gene, with links to repositories that are distributing the mutant mouse strains and the availability of IKMC ES cell resources and the molecular structures of the mutant alleles. Both mutant alleles and phenotype data are seamlessly integrated with existing community resources and databases. For instance, the Mouse Genome Informatics (http://www.informatics.jax.org/) database is used for defining mouse genetic alleles (9) and Ensembl (http://www.ensembl.org/) for defining genomic contexts (10).

In addition to the information made accessible through the web portal, the IMPC computational framework allows public access to the raw data using standard software interfaces and web services. The software application source code for these components and statistical analysis tools are also provided for community use.

## THE IMPC WEB PORTAL

The services provided by the IMPC fulfill the needs of several user groups representing different strands of the biomedical community. These include external use cases 1–5 and project-specific use cases 6–7.

1. The community of biomedical researchers accessing statistically significant phenotypic associations for a given gene, e.g. a rare disease researcher searching for specific phenotypes of interest.
2. Researchers requiring mouse specimens or genetic material for which phenotype data are available, e.g. a researcher who wishes to conduct secondary experiments on a well-known gene to augment existing broad-based phenotype data.
3. System biologists and statisticians seeking access to large-scale standardized gene-phenotype datasets to perform their own analysis.
4. Informatics users accessing all, or partial, datasets for inclusion in their own resource set, e.g. project FaceBase (https://www.facebase.org) (11) that catalogs animal models related to craniofacial abnormalities.
5. High-throughput phenotyping centers producing and exporting their data, e.g. KOMP2 or IMPC members.
6. Data wranglers (dedicated experts in reviewing and QC of phenotypic data) carrying out quality control (QC) checks on the raw data, e.g. correction of data submission errors, detection of baseline drift due to instrumentation, harmonization and standardization of protocols across centers, among others.
7. Funding bodies that are tracking the progress of mouse production and phenotyping efforts, and the state of production, collection and dissemination of the data.

Given the diverse user groups that the IMPC services are targeting, we have created a range of specialist software tools for data analysis and dissemination that caters to these specific requirements. Personas are created for user groups, and extensive usability testing of new interface features is performed. User feedback and testing is gathered via deployment of a beta testing site and is conducted with small groups of users to ensure the components meet their needs. Applications are developed over a unifying framework of distributed computational resources and databases and delivered via the web portal, which we discuss in detail later.

### Data access

The web portal’s primary function is to display genotype–phenotype data for knockout lines for the biomedical community. The site has been optimized to allow free text queries that return structured data via facets allowing the user to explore the data with a mixture of query terms. For example, a query for ‘Pfn1’ returns a summary page with a link to the gene and indicates the production status and availability of phenotype data of mouse strains carrying a null allele of the gene. Users are able to register for genes of interest and will be alerted via email when the gene changes status, indicating new data are available or a mouse is available. A query for a general term ‘glucose’ returns a summary page with a list of matches based on the results for glucose-related genes, phenotyping protocols measuring glucose function and glucose-related phenotypes. Similarly, anatomical queries such as ‘eye’ return results for genes, protocols and phenotypes as well as relevant images indexed by their annotations.

**Figure 1.**
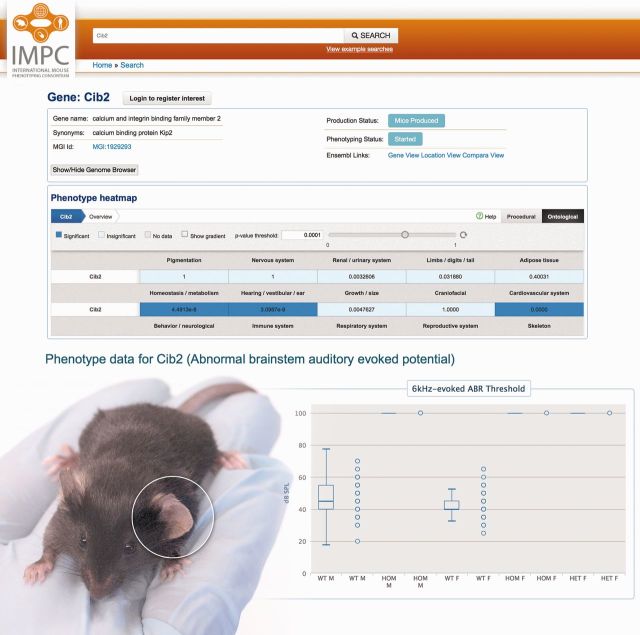
A view of the gene and phenotype data for Cib2, a calcium and integrin-binding family protein. The phenotype heatmap shows significant phenotypes for auditory and brainstem and behavioral tests (*P* < 0.0001). Users can explore underlying data by clicking on phenotype names. The graph shows Cib2 homozygous knockout animals have impaired response to sound stimulus indicating a significant hearing defect as well as abnormal ear morphology. A stock image of an abnormal ear is provided for reference.

**Figure 2.**
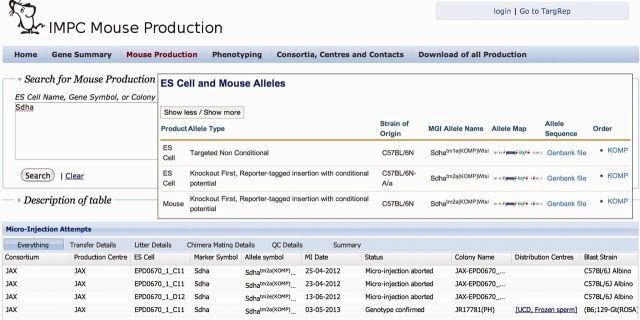
iMITS (https://www.mousephenotype.org/imits) stores and provides summary and detailed production information: Users can view high-level allele information on the IMPC portal gene pages. The iMITS tab of the IMPC portal shows detailed IMPC production information, e.g. for Sdha. Information to access iMITS is provided on the IMPC homepage.

Phenotype data are obtained from the IMPC web portal by several routes that are tailored to the user groups described earlier. Dedicated gene pages contain phenotype association tables that list statistically significant phenotypes that occur in mouse strains carrying null mutations of the given gene (Figure 1). One use case, identified among different types of users, was the need to allow immediate access to data, before a strain has completed phenotyping. Therefore, data are uploaded as soon as they are quality controlled after export from the centers and are available on gene pages with the status ‘IMPC Phenotyping Status Started’. Users can navigate to underlying data supporting these assertions by clicking on graph icons. The gene page also contains a list of ES cells and mice available for this gene, with links to the detailed molecular structure of the allele and links to repositories that distribute the materials (Figure 2).

Phenotype pages present a list of mutant mouse strains associated with a phenotype described using Mammalian Phenotype Ontology terms (12). The assigned terms reflect the phenotyping procedures, which assay anatomy, behavior, blood chemistry, etc of the mutant line. For example, the ‘corneal opacity’ page available at http://www.mousephenotype.org/data/phenotypes/MP:0001314 displays a list of all mouse strains in the database associated with this MP term. A selected representation of the abnormal phenotype is provided when images are available.

### Public access to data and code repository

We have identified user groups requiring programmatic access to data. We provide a Simple Object Access Protocol (SOAP) web service to retrieve the diverse standard operating procedures (SOPs) from the IMPC pipeline and legacy protocols (http://www.mousephenotype.org/impress/soap/server?wsdl) and a RESTful interface to the mouse alleles, experimental results and genotype–phenotype associations from the statistical analysis. We also provide both RESTful and BioMart interfaces to access details of mouse production from the portal (http://www.mousephenotype.org/imits). The portal code is distributed under the Apache v2 software license and available on GitHub (https://github.com/mpi2) and supported by user documentation (https://github.com/mpi2/PhenotypeArchive/wiki). The project uses an agile development approach, and delivers new software releases via the portal and supporting code releases every month.

## DATA ACQUISITION AND QC

Phenotype data produced by the centers are first recorded in local laboratory information management systems managed by the individual phenotyping centers. However, for these data to become part of the IMPC dataset, the data must be captured by rigorously following a well-defined set of SOPs. Furthermore, the recorded measurements must conform to a standardized specification, which includes the unit of measurement, the number of measurements to be taken and other essential metadata. Within the IMPC consortium, phenotyping experiments are referred to as ‘procedures’, and the set of measurements produced by a procedure as ‘parameters’. The SOPs and the specifications for each of the procedures and parameters are stored in the IMPReSS database (see later).

### Phenotype data collection, validation and dissemination

**Figure 3.**
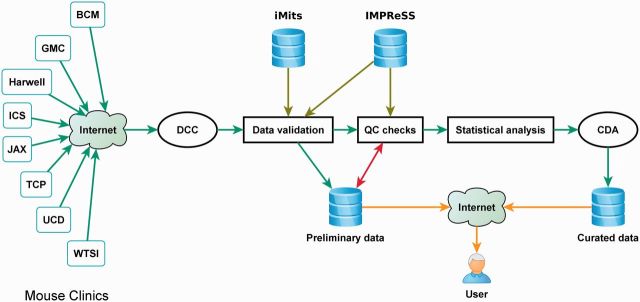
A schematic overview of data flows into the web portal for IMPC data. Currently, eight mouse clinics are involved in IMPC and produce phenotype data. These are then collected, validated and processed to produce curated data available from the project portal. Legacy data from EuroPhenome and Sanger MGP were directly transferred to the Central Data Archive at EMBL-EBI for direct integration on the portal.

When the data are ready for collection and collation, the phenotyping centers export their data as Extensible Markup Language (XML) documents (http://www.w3.org/TR/REC-xml/). These are documents that conform to the standardized data exchange format defined by the IMPC consortium using the XML Schema Definition Language (XSD) specified by the W3C consortium (http://www.w3.org/TR/xmlschema11-1/, http://www.w3.org/TR/xmlschema11-2/). The IMPC Data Coordination Centre at MRC Harwell then downloads these documents. The provenance and chain-of-custody of the data is managed using a data tracker, not presented here. As shown in Figure 3, data processing happens in three main phases to ensure the highest level of data integrity and traceability. In the first phase, the data exported by the mouse clinics are validated against the required procedure and parameter specifications as defined in the SOPs (Data Coordination Centre component), and the supplied values are checked against the corresponding context-specific databases; e.g. check for existence of a mouse strain in the IMPC Mouse Tracking System (iMITS). In the second phase, validated data are incorporated to the centralized dataset, and additional processing is carried out to prepare the data for effective visualization and statistical analysis. The data are then made available to the data wranglers for QC checks and also to researchers for preliminary data analysis. In the third and final stage, data that have passed QC are sent to the Central Data Archive at EMBL-EBI, where they are made available as curated phenotype data. The pipeline is designed to ensure data are publicly available as quickly as possible to the users of the portal.

### Quality control

The IMPC aims to provide the highest quality data to the biomedical community. QC checks are performed in addition to the checks performed at the mouse clinics. The QC process involves identifying anomalies in the submitted data. The aim is to remove data entry and communication errors before the measurements undergo extensive statistical analysis. Some of the QC issues identified are missing data for required parameters, missing wild-type measurements, duplicate measurements, measurements with wrong units, unexpected values (e.g. 0 or negative body weight), out-of-bounds and outliers, among others. These are then communicated to the phenotyping centers, which either fix the issue by correcting the error or provide an explanation. All of the identified issues are captured and managed using custom QC tools. QC tools provide the users (mouse centers and data wranglers) with an integrated workbench for visualization, analysis, identification and resolution of QC issues. By providing an interactive web application that is designed specifically for the visualization of mouse phenotype data, we are able to streamline the workflow.

### Data availability

Phenotype data collection started in early 2013, and to date 2 079 607 data points at eight mouse clinics are available and are undergoing QC before export for archiving (Table 1). This excludes legacy data that are already archived and available for query. Data for 19 different phenotyping procedures are available in the IMPReSS database (Table 2). Mouse production of IKMC alleles has been tracked since 2008. To date, ES cell microinjections that have produced >3000 mouse lines are recorded.

**Table 1.** Mouse phenotyping data points by submitting center, September 2013

Mouse clinics	Number of data points
Baylor College of Medicine	0
Helmholtz Zentrum München	75 662
Institut Clinique de la Souris	446 670
MRC Harwell	164 037
The Jackson Laboratory	142 221
The Toronto Centre for Phenogenomics	20 365
University of California, Davis	59 190
Wellcome Trust Sanger Institute	1 171 462
Total	2 079 607

**Table 2.** Mouse phenotyping data points by SOP, September 2013

Procedures	Number of data points
Acoustic startle and pre-pulse inhibition (PPI)	80 787
Auditory brain stem response	30 606
Body composition (DEXA lean/fat)	59 232
Body weight	205 194
Challenge whole body plethysmography	48 464
Clinical blood chemistry	152 217
Combined SHIRPA and dysmorphology	158 199
Echo	5916
Electrocardiogram (ECG)	35 407
Eye morphology	181 839
Grip strength	71 298
Heart weight	26 520
Hematology	94 416
Indirect calorimetry	627 049
Insulin blood level	38
Intraperitoneal glucose tolerance test (IPGTT)	70 141
Open field	34 416
Organs weight	11 746
X-ray	186 122
Total	2 079 607

## SOPS AND PROTOCOLS

IMPC provides high-quality phenotype data by following rigorous data collection processes. This is achieved by reducing exposure to human error via semi-automated data collection and validation processes. The application of such procedures across multiple centers allows reliable detection of subtle phenotypes, e.g. in the broad-based phenotyping of C57BL/6J and C67BL/6N mouse strains (13). Part of this automation is made possible due to the IMPC protocols, which consist of the SOPs and the procedure and parameter specifications. These protocols are maintained in a form that is both human readable and machine consumable. The IMPC protocols are available from the International Mouse Phenotyping Resource of Standardized Screens (IMPReSS), one of the services provided by the IMPC infrastructure. Machine-readable web services (https://www.mousephenotype.org/impress/soap/server?wsdl) are used in the validation processes discussed in the data acquisition and QC section, and a dedicated relational database stores SOPs.

The adoption of standardized phenotyping protocols across all of the participating mouse clinics requires that the same procedures be carried out under the same conditions specified by the protocol. These protocols have been agreed through active collaboration between the data wranglers (who also administer the contents of the IMPReSS database), the phenotyping centers and members of the scientific community.

The IMPReSS database maintains multiple phenotyping pipelines, where a ‘pipeline’ is simply an ordered sequence of phenotyping procedures to be carried out. This caters to specific circumstances where a center wishes to record and export supplementary data in addition to those that are required by the standard IMPC pipeline. This allows incorporation of data collected using historic pipelines, such as EUMODIC. The IMPReSS database uses the Mammalian Phenotype (MP) Ontology terms (12,14) to annotate procedures and parameters, e.g. the parameter ‘increased blood glucose concentration’ is annotated to ‘impaired glucose tolerance’ (MP:0005203). These ontology terms convert a numerical data point, via statistical analysis and the term annotated to the SOP to provide text definitions of pheno-deviance (a statistically significant result indicating a phenotype different from a wild-type animal of the same background strain). A specific knockout line may have many different terms annotated to fully capture the phenotypes elicited by multiple SOPs, and reflecting the complexity and variety of the SOPs applied.

## DATA INTEGRATION AND ONTOLOGIES

The IMPC portal relies on publicly available data integrated in context for different categories of users. For example, the IKMC resource (4) provides information on ES cells availability, mouse repositories such as EMMA (15) provide access to mice, Ensembl (10) provides the genomic framework for each knockout and Mouse Genome Informatics provides gene nomenclature and mouse ontology terms. Ontologies are widely used throughout the portal. Project-specific views or slims, which provide a relevant subset of the ontology of the Mouse Phenotype Ontology and the Adult Mouse Anatomy Ontology (16), are used to annotate mutants, support online user queries and are built into the schema of our RESTful interface. Ontologies are stored locally in a dedicated part of the schema for ease of query and processing, and we expect to integrate terms mapping human anatomy and disease to data in the future.

## STATISTICAL ANALYSIS

A major goal of the IMPC is to assign functions to protein-coding genes using high-throughput phenotyping assays and to extend the primary observations into specialized fields of research using additional secondary phenotyping screens. High-throughput phenotyping assays produce many different types of data that may be continuous, categorical or time-series numerical data, images or text descriptions of the parameters measured during this assay. Data generated from knockout mice are then subjected to statistical analysis where the parameters measured during the assay are compared with the same parameters measured in parallel from control wild-type mice from an identical background strain. The experimental design also plays a fundamental role in the implementation of a robust and reproducible analysis of knockout phenotypic effects (17,18), which requires control selection to be given considerable attention.

To identify pheno-deviant lines, we have implemented, using the *R* statistical computing toolkit (http://www.r-project.org), a statistical analysis pipeline based on the comparison of each knockout line population with a wild-type control population from a well-defined genetic background (C57BL/6N). Continuous and time-series data are analyzed using a linear mixed model framework (17,19). Linear mixed models multiple sources of variability on a phenotype, where some explanatory factors such as sex, weight and knockout mutant genotype are assumed to take fixed values, while others such as batch (measurements collected on a particular day) will be source of random effect (for example owing to laboratory conditions). We summarize time-series data (e.g. area under the curve, or mean), and this variable is then used into the linear mixed model as a continuous variable. Categorical data contain data separable in mutually exclusive categories and deal with qualitative attributes of the observed object. A Fisher exact test is performed on categorical data and provides a quantitative description of the differences between the knockout and wild-type populations. For each knockout line, we aim to analyze data for seven males and seven females. When a test is considered statistically significant, ontology terms from the Mammalian Phenotype Ontology (12) are automatically associated to the individual genotypes based on association specified in IMPReSS for every parameter (14).

## PROJECT TRACKING

The iMITS (http://www.mousephenotype.org/imits) is the central database for the planning and tracking of IMPC mouse production. The database contains the catalogs of all IKMC ES cell clones and IMPC mouse alleles, their detailed molecular structure and QC data that verify the mutant allele (5). Mutant cells and mice are made available to the scientific community on request via designated repositories. iMITS facilitates the distribution of these products by capturing information on the nominated distribution center(s) and providing appropriate order links. IMPC mouse production centers cooperate to maximize production efficiency and avoid duplication of effort. Each IMPC production center registers the genes selected for production and phenotyping in the iMITS database. Conflicting intentions are flagged. Once an IKMC ES cell clone is microinjected, centers upload details of the microinjection experiments, onward breeding and progress of phenotype data collection and transfer. Actual and intended production is immediately displayed on gene pages in the IMPC portal, and the data are publicly available for browsing and downloading.

Summary iMITS ES allele and mouse production data are displayed on the IMPC portal, and detailed in-progress production information can be found by directly browsing the iMITS Web site (Figure 2). The iMITS infrastructure allows users to be notified by email on the status of the knockout mouse production by registering interest, as described earlier.

## LEGACY DATA

The IMPC portal consolidates data access to existing phenotyping data from the EuroPhenome and Sanger Mouse Genetics Project (MGP) pipelines. Where these data are available for a gene or phenotype of interest, their origin is clearly marked in the interface and links. To date, >11.5 million data points are available for legacy data. Genotype–phenotype associations from EuroPhenome and MGP are presented in Table 3 and classified by high-level mammalian phenotype terms. The inclusion of these data is the key to the mission of the IMPC and the MPI2 consortium, which is to unify access to data, and to provide a stable archive.

**Table 3.** Genotype–phenotype associations from legacy EuroPhenome and Sanger MGP available from the IMPC portal, September 2013

Mammalian phenotype high-level terms	Genotype–phenotype associations
Behavior/neurological phenotype	1268
Homeostasis/metabolism phenotype	1032
Growth/size phenotype	724
Hematopoietic system phenotype	702
Skeleton phenotype	450
Vision/eye phenotype	441
Adipose tissue phenotype	135
Limbs/digits/tail phenotype	125
Craniofacial phenotype	107
Cardiovascular system phenotype	57
Integument phenotype	33
Nervous system phenotype	24
Pigmentation phenotype	20
Immune system phenotype	4
Reproductive system phenotype	4
Endocrine/exocrine gland phenotype	3
Digestive/alimentary phenotype	2
Total	5309

Associations are grouped by high-level mammalian phenotype ontology terms.

## CONCLUSION

The IMPC Web Portal provides unique and unified access to mouse phenotyping data from multiple sources, including genomic, genotypic and phenotypic context from ontologies and the literature and phenotypic images. Access is provided to data as soon as it is available, and for existing legacy data. In future, we will support data access for new embryonic phenotyping pipelines, integrate public gene expression data and make the data more accessible to translational researchers by inclusion of queries for human orthologs, diseases and rare disease data. The statistical pipeline is likely to be refined as more phenotype data are produced and data will regularly be examined to ensure high standards are maintained as new data are submitted. We invite users to register for data of interest via the interface or to sign up for usability, or beta testing activities to improve the portal and provide input into future developments.

## FUNDING

The National Institutes of Health (NIH) [1 U54 HG006370-01]; EMBL-EBI Core Funding (to H.P. and P.F.) and Wellcome Trust Core Funding (to W.C.S.). Funding for open access charge: NIH [1 U54 HG006370-01].

*Conflict of interest statement*. None declared.
